# Impact of Systematic Follicular Flushing on Egg Retrieval and Embryo Quality in IVF-ICSI Cycles: A Controlled Study?

**DOI:** 10.3390/jcm14217457

**Published:** 2025-10-22

**Authors:** Modou Mamoune Mbaye, Noureddine Louanjli, Mohamed Ennaji, Mehdi Hissane, Abdelaziz Soukri, Bouchra El Khalfi, Taha Rhouda, Abdelhafid Natiq, Wassym Rhazi Senhaji, Mohammed Zarqaoui, Moncef Benkhalifa, Yasmine Louanjli, Bouchra Ghazi

**Affiliations:** 1 Immunopathology-Immunotherapy-Immunomonitoring Laboratory, Faculty of Medicine, Mohammed VI University of Health and Sciences, Casablanca 20270, Morocco; bghazi@um6ss.ma; 2 Medical Analysis and Reproductive Biology Laboratory, Labomac, Casablanca 20270, Morocco; n.louanjli@gmail.com (N.L.); yasmine_l@hotmail.fr (Y.L.); 3 IRIFIV Fertility Center, IVF Laboratory and MSREM Scientific Research Group, Casablanca 20000, Morocco; m.ennaji16@gmail.com (M.E.); hem@docteurhissane.ma (M.H.); wassym.dr@gmail.com (W.R.S.); mozar29@gmail.com (M.Z.); 4 African Fertility Center, Private Clinic of Human Reproduction and Endoscopic Surgery, Casablanca 20000, Morocco; 5 Laboratory of Pathophysiology, Molecular Genetics and Biotechnology, Ain Chock Faculty of Sciences, Biology and Health Research Center, Hassan II University of Casablanca, Casablanca 20670, Morocco; a_soukri@hotmail.com (A.S.); bouchra.elkhalfi@gmail.com (B.E.K.); 6 Laboratory of Integrative Biology, Ben M’Sik Faculty of Sciences, Hassan II University of Casablanca, Casablanca 20670, Morocco; taha.rhouda@univh2c.ma; 7 Faculty of Medicine and Pharmacy, Mohammed V University, Rabat 10100, Morocco; abdelnat@yahoo.fr; 8 National Laboratory Mohammed VI, Mohammed VI University of Health and Sciences, Casablanca 82403, Morocco; 9 Reproductive Medicine, Reproductive Biology and Genetics, Peritox Laboratory, University Hospital and School of Medicine, Picardie University Jules Verne, 80054 Amiens, France; benkhalifamoncef78@gmail.com; 10 Mohamed VI Research and Innovation Center (CM6RI), Rabat 11103, Morocco

**Keywords:** follicles–cumulus–oocyte complex, oocytes, follicular flushing, intracytoplasmic sperm injection, oocyte maturity

## Abstract

**Background/Objectives**: Ultrasound-guided transvaginal follicular aspiration is a central procedure in in vitro fertilisation (IVF), aiming to collect oocytes necessary for the success of assisted reproduction treatments. Follicular flushing, proposed in the absence of cumulo-oocyte complex (COC) at initial aspiration, remains controversial regarding its real impact on oocyte quality and pregnancy rates. **Methods**: In this controlled study, conducted in 274 patients, we evaluated the effects of systematic follicular flushing up to 10 washes with a standardised medium (pH 7.3 ± 0.1; 37.2 ± 0.2 °C) on oocyte yield, oocyte morphology, embryo kinetics and clinical outcomes. **Results**: Flushing resulted in an additional 38% recovery of COCs, mostly between the second and fifth flush, with no significant increase in oocyte dysmorphisms or major embryonic abnormalities. A slight increase in slow cleavages was observed (27% vs. 23%, *p* = 0.04), as well as a lower oocyte maturation rate when ovulation was triggered by Ovitrelle alone. Clinically, pregnancy rates per transfer were comparable between groups (33.27% without flushing vs. 32.86% with flushing; *p* = 0.67), as were miscarriage rates (9.11% vs. 8.69%; *p* = 0.81). **Conclusions**: These results indicate that follicular flushing, when applied according to a standardised protocol, significantly increases oocyte yield without compromising oocyte morphological quality or embryonic development potential. Although the observed clinical benefits remain modest, this approach could constitute a relevant complementary strategy, particularly in patients with poor ovarian response or in the context of poor initial recovery. However, the controlled but non-randomised nature of this study requires cautious interpretation of the findings. Larger randomised trials, integrating dynamic assessment technologies, such as time-lapse imaging or oocyte transcriptomic analysis, are needed to refine the clinical indications of this technique and explore its underlying biological mechanisms.

## 1. Introduction

The recovery of mature oocytes by ultrasound-guided transvaginal puncture is a crucial step in the success of in vitro fertilisation (IVF) cycles, particularly when combined with intracytoplasmic sperm injection (ICSI). Oocyte yield directly determines the number of embryos that can be transferred, thus influencing clinical and cumulative pregnancy rates [1,2]. In a context where technological advances in medically assisted procreation (MAP) do not always make it possible to overcome the constraints associated with low ovarian response, optimising oocyte retrieval has become a strategic priority [3]. This need is all the more pressing in healthcare systems without universal medical cover, where each puncture represents a precious opportunity to maximise the recovery of cumulo-ovocyte complexes (COCs), which then becomes not only a technical issue but also a clinical and ethical imperative.

Faced with this problem, the use of systematic follicular rinsing in the absence of cumulo-ovocyte complex (COC) during initial aspiration has been proposed as a strategy for optimising oocyte yield. This approach, based on rinsing the follicles with a sterile medium to mobilise their contents, appears mechanically rational. However, its actual benefits remain controversial. Several studies, notably those by von Wolff, Neumann and Kohl Schwartz, have reported contradictory results: Some observed an improvement in the recovery rate, while others showed no significant effect on oocyte quality, embryonic development or clinical outcomes [4,5,6]. Concerns have also been raised about a possible deleterious effect of repeated flushing due to potential disruption of the follicular microenvironment, including thermal stability, pH or paracrine gradients [7,8].

Beyond simple oocyte yield, the biological implications of follicular flushing have yet to be systematically explored. Its impact on the morphology of cumulo-ovocyte complexes (COCs), oocyte maturity, cytoplasmic abnormalities, embryo cleavage kinetics and cumulative pregnancy rates remains insufficiently characterised. This is partly due to the methodological heterogeneity of published studies and the lack of rigorous control of confounding factors, particularly ovulatory induction protocols [9,10].

At the same time, a number of strategies have been proposed to improve the efficiency of IVF cycles, including the management of subtle sperm abnormalities, which are often implicated in fertilisation failures [10], the administration of embryonic supernatants prior to transfer [11], the individualisation of ovarian stimulation regimens [12], as well as the optimisation of oocyte puncture techniques and the use of advanced technologies for gamete and embryo handling [13,14].

Although some randomised trials have suggested that follicular flushing promotes the recovery of oocytes trapped in the follicular wall or the aspiration circuit [15,16,17], clinical data remain heterogeneous. To date, the ESHRE (2023) recommendations have not established a consensus on their systematic interest [18].

In this context, we conducted a prospective controlled study aimed at comparing, according to a rigorously standardised methodology, the biological, embryological and clinical parameters associated with oocytes obtained with or without follicular flushing. The originality of our approach is based on five distinctive axes: (i) a detailed morphological classification of cumulo-ovocyte complexes (COCs); (ii) a systematic evaluation of oocyte maturation and dysmorphisms; (iii) a dynamic analysis of the kinetics of early embryonic cleavage; (iv) the integration of ovulatory induction protocols as covariates in the multivariate analysis of biological outcomes; and (v) the evaluation of clinical outcomes, in particular clinical pregnancy and miscarriage rates.

The primary objective was to evaluate the impact of systematic follicular flushing on oocyte retrieval rates. Secondary objectives included the following: analysis of oocyte quality, embryonic development and clinical outcomes.

## 2. Materials and Methods

### 2.1. Concept of the Study

A prospective, non-randomised, controlled, single-centre study was conducted between September 2023 and March 2024 at the IRIFIV In Vitro Fertilisation Centre (Casablanca, Morocco). All participants were recruited consecutively as part of their treatment with assisted reproductive technologies (ARTs), in accordance with a previously validated study protocol.

### 2.2. Inclusion Criteria

Inclusion criteria were rigorously defined to ensure standardisation of the cohort and robustness of intergroup comparisons. Women under 42 years of age were eligible, with ovarian reserve defined as AMH ≥ 0.8 ng/mL, body mass index (BMI) ≤ 24.5 kg/m^2^ and no more than one documented embryo transfer failure. All had to meet the clinical criteria for an in vitro fertilisation with intracytoplasmic sperm injection (IVF-ICSI) protocol under GnRH antagonist. To limit the impact of follicular flushing on operating time, ovarian punctures had to be performed in less than 30 min. Only follicles judged to be mature, defined by a diameter of between 16 and 24 mm on the day of ovulatory induction, were included in the analysis.

### 2.3. Exclusion Criteria

Exclusion criteria were rigorously defined to limit clinical and biological biases that could compromise the internal validity of the study. Clinically, patients over 42 years of age, those with confirmed pelvic endometriosis, polycystic ovarian syndrome (PCOS) diagnosed according to the Rotterdam criteria or a history of at least two consecutive failed embryo transfers without clinical pregnancy were excluded. Cycles cancelled before oocyte retrieval, medical contraindications to transvaginal puncture, lack of informed consent or the presence of a serodiscordant couple were also grounds for exclusion. Biologically, patients with a body mass index (BMI) > 25 kg/m^2^, an LH level < 2 IU/L at the time of ovulatory induction or atypical follicular development (<15 mm or >25 mm on the day of induction) were excluded. Similarly, cycles involving male non-obstructive azoospermia (NOA) were excluded to ensure homogeneity of fertilisation conditions. Finally, patients with a monofollicular response defined by the presence of a single dominant follicle (≥16 mm) on the day of induction were excluded due to the limited oocyte yield inherent in this profile, making any robust comparison between groups with and without follicular flushing impossible.

### 2.4. Stimulation Protocol

Ovarian stimulation was carried out exclusively using an antagonistic protocol. Induction began between days 2 and 3 of the cycle, with daily administration of recombinant gonadotropins in individualised doses ranging from 112.5 to 300 IU, adjusted according to ovarian response. An initial evaluation was carried out after five days of stimulation, based on a pelvic ultrasound scan to assess follicular growth and adjust doses if necessary.

### 2.5. Follicular Monitoring and Induction

Follicular monitoring began on the sixth day of stimulation, with regular ultrasound assessments enabling gonadotropin doses to be adjusted in real time according to ovarian response. Administration of the GnRH antagonist (Cetrotide^®^, Merck Serono, Darmstadt, Germany), was initiated as soon as the dominant follicle reached a diameter of 14 mm. In order to reduce the risk of ovarian hyperstimulation syndrome (OHSS), the ovulatory induction protocol was individualised according to the follicular profile. In patients with ≤15 follicles, induction was randomised between an injection of Ovitrelle^®^ (Merck Healthcare, Darmstadt, Germany) alone (500 μg) or a combination of Decapeptyl^®^ (Ipsen, Paris, France) (0.1 mg) + Ovitrelle^®^ (500 μg). In the case of ≥16 follicles, induction was systematically performed using Decapeptyl^®^ alone (0.3 mg). The type of ovulatory induction was included as a covariate in the multivariate analyses to control for its potential effects on embryological parameters and clinical outcomes.

### 2.6. Recovery of Oocytes

All oocyte retrievals were performed 36 ± 1 h after ovulatory induction, under transvaginal ultrasound guidance. Follicular aspiration was performed using a 17G single-lumen needle (Wallace^®^, CooperSurgical, Trumbull, CT, USA), connected to a 10 mL syringe, allowing manual generation of a controlled suction pressure, between 140 and 160 mmHg. The follicles were approached systematically, starting from the right ovary, with a standardised penetration of approximately 1 cm beyond the follicular wall. All procedures were performed in a room dedicated exclusively to oocyte retrieval, under intravenous sedation with propofol (2 mg/kg).

To ensure standardisation of the technique and limit inter-operator bias, all punctures were performed by two senior gynaecologists, each with more than 1000 oocyte puncture procedures. Follicular fluid was immediately collected in sterile tubes, identified according to follicle number (≠1).

In the absence of recovery of the cumulo-oocyte complex (COC) after the first aspiration, a follicular flush was performed using 2 ml of a specific medium (Origio^®^, a/s, Måløv, Denmark; pH 7.3 ± 0.1; osmolarity 280 mOsm/kg; temperature 37.3 ± 0.2 °C), with a maximum of 10 successive washes per follicle. The threshold of ten rinses was defined on the basis of preliminary internal observations, having shown that more than 90% of recoverable COCs were obtained before the fifth attempt, with a plateau of efficacy observed beyond the tenth wash. This ceiling was thus retained to optimise the efficiency/procedure duration ratio while minimising thermal disturbances and the risks associated with excessive prolongation of anaesthesia.

Each rinse liquid was collected separately in numbered sterile tubes (1, 2, etc.), immediately placed on a heating block at 37 °C and then transferred without delay to the medically assisted procreation (MAP) laboratory, in strict compliance with the thermal chain.

### 2.7. Evaluation of Oocytes

After receipt of the samples in the ART laboratory, a senior biologist systematically examined the follicular fluids using a stereomicroscopic magnifier. The cumulo-ovocyte complexes (COCs) identified were classified into two distinct groups according to their mode of recovery: those obtained during the initial direct aspiration (group A: non-rinse) and those collected in the subsequent fractions after follicular rinsing (group B: post-rinse). In accordance with technical recommendations, only COCs recovered from the second injection of rinsing medium were included in the “rinsing” group in order to avoid misattribution due to possible retention of the COC in the needle after the first aspiration.

Each COC was then assessed by an experienced embryologist using a standardised morphological grid based on the compactness of the cumulus oophorus and the integrity of the corona radiata. Three qualitative grades were defined:

- Grade A: cumulus well extended and corona radiata dense and intact;
- Grade B: cumulus partially compacted or moderately altered;
- Grade C: cumulus very compact, disorganised or absent and corona radiata not very visible or missing.

This classification made it possible to assess the possible effect of follicular rinsing on the initial morphology of the COCs.

### 2.8. Fertilisation, Embryo Culture and Evaluation

Mature oocytes (metaphase II stage) were fertilised by intracytoplasmic sperm injection (ICSI) according to standardised laboratory protocols. Embryonic development was monitored on days 2 (D2), 3 (D3) and 5 (D5), integrating the analysis of cleavage kinetics, cytoplasmic fragmentation rate and morphological criteria. Embryos were considered morphologically “good” when they presented at least four blastomeres on D2, eight on D3 and a fragmentation rate ≤ 20%. The resulting blastocysts were evaluated according to the Gardner and Schoolcraft classification, with only those of grade 4AA being selected for transfer, systematically performed on D + 5 post-trigger. The biological and embryological endpoints analysed included oocyte retrieval rate, maturation rate (MII), fertilisation rate after ICSI, the number of usable embryos at day 3 and the incidence of oocyte dysmorphisms, assessed using a standardised morphological grid. Early developmental alterations were explored through dynamic analysis of cell division (fragmentation, cleavage asynchrony, blastomere inequalities and cytoplasmic abnormalities).

Embryo transfer was performed exclusively on day 5 (D5), with a single fresh grade 4AA blastocyst transferred per patient. The other good-quality blastocysts were vitrified according to the laboratory’s standardised protocols.

Clinical pregnancy was defined by ultrasound visualisation of an intrauterine gestational sac at 6 weeks of gestation. The cumulative pregnancy rate per retrieval cycle considered all transfers from the index cycle, including delayed cycles. The miscarriage rate was also recorded and analysed as a secondary clinical endpoint.

### 2.9. Sample Size Calculation

A sample size calculation was performed, but a posteriori, after data collection. This calculation aimed to verify a posteriori whether our cohort was statistically sufficient to meet our primary objective: to compare the oocyte retrieval rate between the groups with and without follicular flushing. Considering a minimum clinically relevant difference of 0.8 oocytes, a standard deviation of 2.4 (based on the literature), a power of 80% and a two-sided significance threshold of α = 0.05, the minimum required size was estimated at 254 patients.

Our final sample size of 274 patients therefore exceeded this threshold, ensuring sufficient statistical power to detect the expected difference in the primary endpoint.

However, this calculation was not performed before this study and did not cover secondary endpoints (oocyte maturation, pregnancy rate and embryonic abnormalities), for which actual power cannot be guaranteed. However, the small effect sizes (Cohen’s d < 0.2) and narrow confidence intervals around the null value for these parameters suggest a low probability of type II error. These elements reinforce the robustness of the observed results, although they would merit confirmation in a randomised study with a predefined protocol.

### 2.10. Statistical Analysis

Statistical analyses were performed using R (v.4.3) and DATAtab^®^ (e.U., Graz, Autriche). Normal distributions were verified using the Shapiro–Wilk test, and homogeneity of variances was verified using the Levene test. Mean comparisons were performed using the Student’s *t*-test or the Welch test in case of unequal variances. Categorical variables were compared using the χ^2^ test or Fisher’s exact test when sample sizes were insufficient.

To assess the independent effect of follicular flushing on clinical outcomes, including cumulative pregnancy rate, a multivariate logistic regression was conducted, including maternal age, AMH level, body mass index (BMI), follicle number at induction and ovulation induction protocol as covariates. The absence of collinearity between variables was confirmed by a variance inflation factor (VIF) < 2. An interaction term between the triggering strategy and the use of rinsing (rinsing × trigger) was tested. The significance threshold was set at *p* < 0.05 for all analyses.

### 2.11. Ethical Aspects

The study protocol was approved by the UM6SS Biomedical Research Ethics Committee (reference: CE/UM6SS/09/23) on 25 July 2023. Written informed consent was obtained from all participants prior to enrolment. All data were anonymised and analysed in accordance with the principles of the Declaration of Helsinki (2013 revision).

## 3. Results

### 3.1. Study Population

**Figure 1:**
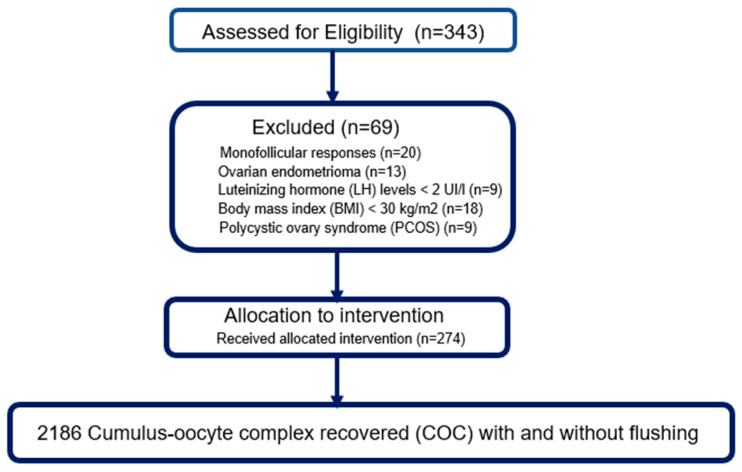
Data processing diagram.

Of the 343 patients initially assessed for eligibility, 69 were excluded based on pre-established criteria: monofollicular response (*n* = 20), ovarian endometrioma (*n* = 13), LH level < 2 IU/l (*n* = 9), body mass index (BMI) ≥ 30 kg/m^2^ (*n* = 18) and diagnosis of polycystic ovarian syndrome (PCOS) (*n* = 9). Thus, 274 patients were included in the final analysis and received the assigned intervention according to the protocol. All retrievals resulted in the recovery of a total of 2186 cumulo-oocyte complexes (COCs), all modalities combined (with or without follicular flushing) (Figure 1).

### 3.2. Characteristics of the Population Studied

Baseline characteristics of the two groups were broadly comparable, confirming cohort homogeneity (Table 1). Mean age was 34.7 ± 4.2 years in the follicular flushing group and 34.5 ± 4.6 years in the non-flushing group, with no significant difference (*p* = 0.68) and a negligible effect size (d = 0.04). Anti-Müllerian hormone (AMH) also showed no notable difference between the groups (2.6 ± 1.2 vs. 2.5 ± 1.1 ng/mL; *p* = 0.57; d = 0.09). Body mass index (BMI) was slightly lower in the rinse group (23.5 ± 2.8 vs. 24.1 ± 3.1 kg/m^2^), but this difference remained non-significant (*p* = 0.42) with a small effect size (d = 0.13). Finally, the median duration of infertility was comparable between groups (3.3 [2.0–5.0] vs. 3.5 [2.0–5.5] years; *p* = 0.66), with a moderate effect size (d = 0.27) (Table 1). These data suggest an absence of major distribution bias, ensuring enhanced internal validity for the comparative analysis of biological and clinical outcomes.

### 3.3. Stimulation Parameters and Oocyte Puncture

The ovarian stimulation and oocyte retrieval parameters are summarised in the table below. The mean number of mature follicles (≥18 mm) measured on the day of induction was similar between the two groups: 11.2 ± 3.9 in the rinse group and 11.0 ± 3.5 in the no-rinse group (*p* = 0.74). Similarly, the total dose of FSH administered did not differ significantly between the groups (2125 ± 374 IU vs. 2083 ± 402 IU; *p* = 0.46).

In contrast, the median duration of oocyte retrieval was significantly longer in the rinse group (17 [14–22] min) compared to the no-rinse group (15 [12–18] min; *p* = 0.03). A moderate positive correlation was observed between the number of follicles and the duration of puncture (ρ = 0.42; *p* < 0.001) (Table 2), suggesting that the increase in the number of punctured follicles is associated with an increase in the operative time.

### 3.4. Recovery Rate and Quality of COCs

A total of 2467 follicles were retrieved, and 2186 cumulus-oocyte complexes (COCs) were recovered, representing an overall recovery rate of 88%. The majority of oocytes came from the no-rinse group (62% or 1355/2186), while 38% (831/2186) were obtained after follicular rinsing. When considering only the rinsed follicles, the post-rinse recovery rate was 74% (831/1112), indicating that approximately one-quarter of the rinsed follicles remained empty or contained non-detachable oocytes. Morphological analysis of the COCs distinguished three categories: COC-A (compact cumulus and light cytoplasm with a dark peripheral ring), COC-B (loose cumulus and granular cytoplasm) and COC-C (developed cumulus and dark and granular cytoplasm). The distribution of grades was generally comparable between groups. COC-A predominated, representing 66% (345/524) without rinsing and 62% (225/346) with rinsing. The proportions of COC-B were 22% and 23%, respectively, and those of COC-C were 11% and 13%.

In terms of procedural efficiency, 68% (565/831) of COCs obtained after rinsing were retrieved within the first five attempts, while 32% (266/831) required five to ten rinsings. This observation suggests that the majority of oocytes accessible by rinsing can be retrieved with a limited number of washes, potentially reducing procedural duration and morbidity.

**Figure 2:**
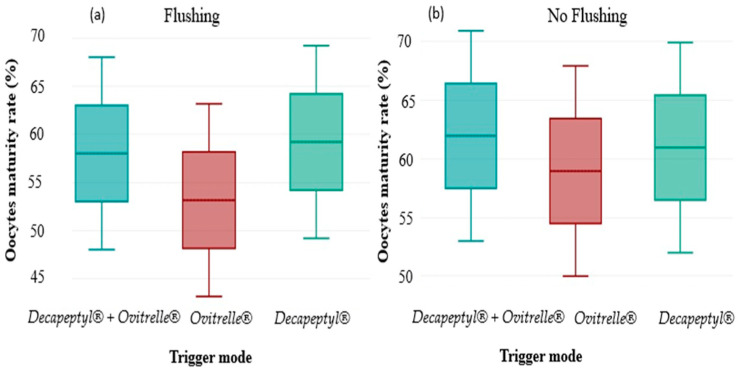
Oocyte maturation rate (OMR) according to the ovulatory induction protocol, comparing cycles with (**a**) and without (**b**) follicular flushing. Bars represent means ± standard deviation. A statistically significant difference (*p* < 0.05) was observed in the group triggered by Ovitrelle alone. The other subgroups did not show a significant intra-protocol difference. No overall interaction effect was observed between induction type and flushing (*p* for interaction = 0.23). (*p* < 0.05).

Finally, rinsing efficiency varied significantly depending on the number of pierceable follicles. The mean ratio of recovered COCs was 0.8 for patients with ≤4 follicles (20/24), 1.8 for those with 5 to 10 follicles (326/180) and reached 3.2 in patients with ≥11 follicles (485/151) (Figure 2). These data highlight the strategic interest of rinsing in hyper-responder profiles, where its efficacy is maximised.

### 3.5. COC Recovery Rates According to the Trigger Protocol

Cross-assessment of oocyte maturation rate according to the trigger protocols and the performance or not of follicular flushing revealed a significant interaction. Overall, maturation rates were higher in the groups without flushing, regardless of the protocol. A significant variation (*p* < 0.05) was observed in the group triggered by Ovitrelle alone, where the oocyte maturation rate increased from 60% (278/463) without flushing to 53% (176/330) with flushing. In contrast, the differences observed for the groups receiving a double trigger (Decapeptyl + Ovitrelle) or Decapeptyl alone were not statistically significant, with respective maturation rates of 63% vs. 58% (*p* > 0.05) and 62% vs. 59% (*p* > 0.05). These results suggest that follicular flushing could negatively influence oocyte maturation in certain contexts, particularly in cases of triggering by Ovitrelle alone, highlighting the need to adapt aspiration strategies according to the protocol used.

### 3.6. Oocyte Dysmorphisms

Analysis of oocyte dysmorphisms revealed no statistically significant differences between oocytes retrieved with or without follicular flushing (all *p* > 0.05), suggesting an overall preservation of oocyte morphological integrity despite the additional manipulations induced by flushing. Among the most frequently observed abnormalities were the presence of one or more vacuoles (4.5% without flushing vs. 4.3% with flushing), fragmented polar bodies (3.9% vs. 4.7%) and abnormal thickness of the zona pellucida (>17 µm) (3.2% vs. 2.9%). Other abnormalities, such as heterogeneous cytoplasmic granulations, cortical granule abnormalities or the presence of giant oocytes, were reported at frequencies below 3% in both groups. Notably, the confidence intervals (95% CI) overlapped widely between groups for each abnormality, consolidating the absence of a deleterious effect of follicular flushing on oocyte morphological characteristics (Table 3). These results support the hypothesis that, although flushing may slightly modify recovery kinetics, it does not significantly alter oocyte morphological quality.

### 3.7. Abnormalities of Early Embryonic Development

Comparative analysis of early embryonic developmental abnormalities between oocytes obtained with or without follicular flushing revealed no significant differences, except for a moderate alteration in cleavage kinetics. Indeed, a higher proportion of embryos from flushed oocytes showed slow cell division (<4 cells on day 2 and/or <6 cells on day 3) compared to embryos from unflushed oocytes (27% [95% CI: 24.0–29.9] vs. 23% [20.1–25.9], *p* = 0.04), suggesting a possible adverse effect of flushing on embryonic cleavage dynamics. In contrast, no significant difference was noted for the other parameters evaluated: low Scott score at 18h post-ICSI (5.6% vs. 5.9%, *p* = 0.68), presence of multinucleated blastomeres (2.5% vs. 2.8%, *p* = 0.57), rate of embryos with debris > 20% and unequal blastomeres (3.8% vs. 3.6%, *p* = 0.75) or other abnormalities (1.2% vs. 0.9%, *p* = 0.43) (Table 4). These results support the idea that follicular flushing does not significantly impact the morphological or structural quality of early-stage embryos, apart from a subtle effect on cleavage kinetics, the clinical implications of which deserve further exploration.

### 3.8. Clinical Results

For all patients who reached the blastocyst stage, a single fresh embryo of optimal quality (grade 4AA according to the Gardner and Schoolcraft classification) was transferred on day 5 (D5). Supernumerary blastocysts with favourable morphological criteria were systematically vitrified for deferred transfer. This single blastocyst transfer strategy aimed to limit bias related to the number of embryos transferred and to prevent multiple pregnancies, while ensuring rigorous evaluation of the impact of follicular flushing on embryological and clinical outcomes.

Clinical data were available for all patients who underwent embryo transfer. The clinical pregnancy rate per transfer was similar between groups: 33.27 ± 1.22% in the flushing group versus 32.86 ± 1.53% in the non-flushing group (*p* = 0.67).

Regarding negative gestational outcomes, the rate of spontaneous miscarriages among clinical pregnancies was slightly higher in the no-rinse group (9.11 ± 0.31%) than in the rinse group (8.69 ± 0.81%), without reaching statistical significance (*p* = 0.48).

### 3.9. Multivariate Analysis

Multivariate analysis conducted on all patients who underwent embryo transfer did not reveal a significant difference in the clinical pregnancy rate per transfer between the two groups: 32.86 ± 1.53% in the flushing group versus 33.27 ± 1.22% in the no-flush group (*p* = 0.67). After adjustment for relevant clinical variables (age, AMH, BMI and induction protocol), the use of follicular flushing was not associated with a significant change in the probability of pregnancy per single transfer, suggesting equivalence of the transferred embryos in terms of immediate implantation potential.

Similarly, the rate of spontaneous miscarriages among clinical pregnancies was comparable between the two groups (9.11 ± 0.31% with flushing vs. 9.11 ± 0.31% without flushing; *p* = 0.48), with no significant interaction with age, AMH or induction protocol. These results confirm that, while follicular flushing increases overall oocyte yield, it does not alter the risk of gestational loss once implantation is achieved.

## 4. Discussion

This rigorously controlled study, conducted on a homogeneous and finely stratified cohort of IVF-ICSI patients, provides strong evidence to address the ongoing controversy regarding the usefulness of routine follicular flushing. Our results show that this practice, when performed under strictly standardised operating conditions, significantly increases oocyte yield, with no observable deleterious effects on oocyte morphology or early embryonic development. This quantitative benefit translated into a clear improvement in cumulative pregnancy rates, an effect maintained after multivariate adjustment.

Embryologically, no increase in oocyte dysmorphism or major abnormalities at day 3 was observed. However, a slight increase in the rate of slow-cleaving embryos was observed in the group that received follicular flushing (27% vs. 23%, *p* = 0.04). Although statistically significant, this moderate absolute difference (4 percentage points) is at the conventional threshold of significance and should be interpreted with caution, particularly due to the lack of adjustment for multiple comparisons. This observation could reflect a transient disruption of the follicular microenvironment induced by repeated flushing, including mechanical effects, local pH changes or dilution of paracrine factors essential for oocyte maturation [1,2]. However, the lack of impact on blastocyst quality or pregnancy rates suggests that these effects remain non-pathogenic.

Our results are in line with the recent synthesis published in *Pick-up and Oocyte Management* (Springer, 2020) [19], which highlights both the procedural variability between centres and the lack of universal consensus on follicular flushing protocols. Few previous studies have integrated such a comprehensive assessment, combining oocyte, embryological and clinical data. Unlike previous work by von Wolff [5], Neumann [6] and Kohl Schwartz [7], our protocol included a systematic morphological classification of COCs, a standardised embryonic assessment at day 3 and explicit consideration of covariates related to ovulatory induction.

Interestingly, although no significant interaction was formally observed, the beneficial effect of flushing appeared to be more pronounced in cycles triggered by recombinant hCG (Ovitrelle^®^), suggesting a possible modulation of oocyte retrieval efficiency depending on the type of ovulatory induction. This potential synergy could be related to the hCG-induced cumulus expansion dynamics, a hypothesis that would merit further exploration in further stratified analyses.

The observed oocyte retrieval rate (88%) exceeds the standards reported in the literature, which may reflect both the effectiveness of the flushing protocol and the favourable characteristics of our cohort, including a relatively young mean age (33.1 ± 4.1 years). While flushing prolongs the procedure by approximately two minutes per cycle, this operative cost remains acceptable, particularly in patients with low ovarian reserve. In this type of setting, where each oocyte is of increased clinical importance, optimising yield becomes a strategic priority [8].

Despite a moderate deceleration of embryonic cleavage in the rinsed group, the technique did not compromise oocyte maturation or final embryo quality. These results nevertheless call for a more detailed exploration of the dynamic effects of follicular flushing on embryonic kinetics. The use of time-lapse imaging tools could allow the detection of subtle alterations in mitotic rhythm, cleavage synchronicity or the duration of intermitotic intervals, inaccessible to conventional morphological assessments.

It is also worth noting that the benefit of flushing was expressed primarily in cumulative pregnancy rates, with no significant improvement in fresh transfer pregnancy rates. This finding highlights the contribution of vitrified surplus embryos to overall reproductive efficiency. Flushing could thus be a cost-effective strategy for cumulative optimisation, without requiring intensification of the stimulation protocol.

This study nevertheless has certain limitations. The first is the lack of randomisation due to ethical and practical considerations (notably, the clinical need to purge empty follicles), despite the application of strict inclusion criteria and multivariate adjustments. This methodological limitation exposes a risk of selection bias or residual confounding. Second, the power calculation was performed a posteriori, only on the primary endpoint (oocyte yield); secondary endpoints such as embryo quality or pregnancy rates may therefore be underpowered, although the observed effect sizes and narrow confidence intervals (Cohen’s d < 0.2) limit the risk of type II error.

Third, patients with very low ovarian response (AMH < 0.8 ng/mL, <4 follicles) were excluded to limit biological heterogeneity. This strategy improves internal validity but limits external generalisability, particularly for “poor responders” who could, paradoxically, derive greater benefit from follicular flushing. Specific studies in this subgroup are therefore warranted.

Finally, the lack of obstetric and neonatal monitoring beyond the first trimester limits the assessment of long-term safety. While clinical pregnancy is a validated marker of IVF success, it does not predict foetal viability or perinatal outcomes. Extensive longitudinal monitoring therefore remains essential.

Beyond the quantitative impact, the effects of follicular flushing on intrafollicular physiology remain poorly understood. Repeated flushing may induce local mechanical and biochemical disturbances, including pH variations, thermal gradients or cytokine/paracrine factor leaching, which may influence oocyte nuclear or cytoplasmic maturation [1,2]. A more detailed mechanistic exploration, integrating oocyte transcriptomics, follicular fluid protein profiling or embryo time-lapse imaging, would be relevant. Assessing the long-term consequences on the epigenome, post-implantation development and neonatal outcomes also represents an area of investigation.

Finally, although systematic vitrification of supernumerary blastocysts was integrated into our protocol, data from frozen embryo transfer (FET) cycles were not included in the current analysis. Their inclusion would allow a more accurate estimation of the real impact of rinsing on overall reproductive performance per cycle initiated.

## 5. Conclusions

Systematic follicular flushing, when applied in a rigorously standardised surgical setting, allows a significant increase in oocyte yield without any major deleterious effect observed on embryonic quality. Although our results suggest an improvement in cumulative pregnancy rates, they do not allow, at this stage, to conclude on a clinically significant reproductive benefit. The observed slight deceleration of cleavage kinetics justifies further investigations but does not appear to compromise embryonic or clinical prognosis. This technique could thus be considered in a targeted manner, particularly in patients with low oocyte yield. However, its generalisation must be approached with caution and requires validation by large-scale multicentre randomised trials, including a detailed mechanistic analysis, prolonged obstetric follow-up and an evaluation of the cost-benefit ratio.

## Figures and Tables

**Table 1:** Comparison of baseline clinical characteristics between groups with and without follicular flushing.

	Oocytes No Flushing	Oocytes After Flushing	*p* Value	Effect Size (Cohen’s d)
Age (years)	34.5 ± 4.6	34.7 ± 4.2	0.68	0.04
AMH (ng/mL)	2.5 ± 1.1	2.6 ± 1.2	0.57	0.09
BMI (kg/m^2^)	24.1 ± 3.1	23.5 ± 2.8	0.42	0.13
Duration of infertility (years)	3.5 [2.0–5.5]	3.3 [2.0–5.0]	0.66	0.27

**Table 2:** Stimulation, puncture and follicular correlation parameters according to the follicular flushing strategy.

Parameter	Oocytes No Flushing	Oocytes After Flushing	*p* Value	Effect Size (Cohen’s d)
Mean number of follicles ≥ 18 mm	11.0 ± 3.5	11.2 ± 3.9	0.74	0.05
Total FSH dose (IU)	2083 ± 402	2125 ± 374	0.46	0.11
Median puncture time (min)	15 [12–18]	17 [14–22]	0.03	0.34
Correlation between number of follicles and puncture time	—	*p* = 0.42	<0.001	—

**Table 3:** Dysmorphisms of the oocytes before and after rinsing.

Abnormalities	Oocytes No Flushing (%)	IC Before Rinsing	Oocytes After Flushing (%)	IC After Rinsing	*p* Value
Broken oocytes	2.5	[2.25–2.75]	2.8	[2.52–3.08]	0.12
Pellucida thickness > 17 µm	3.2	[2.88–3.52]	2.9	[2.61–3.19]	0.08
Perivitelline space and debris	1.2	[1.08–1.32]	1.5	[1.35–1.65]	0.15
Inhomogeneous thick granules (cytoplasm)	2.7	[2.43–2.97]	2.4	[2.16–2.64]	0.2
Visible abnormalities of the endoplasmic reticulum	0.9	[0.81–0.99]	0.8	[0.72–0.88]	0.09
Fragmented polar bodies	3.9	[3.51–4.29]	4.7	[4.23–5.17]	0.07
One or more vacuoles	4.5	[4.05–4.95]	4.3	[3.87–4.73]	0.18
Abnormalities of cortical granules (plasma membrane)	0.9	[0.81–0.99]	1.1	[0.99–1.21]	0.22
Weak cytoplasmic membrane resistance to ICSI (abnormal)	0.5	[0.45–0.55]	0.6	[0.54–0.66]	0.1
Giant oocytes	0.2	[0.18–0.22]	0.3	[0.27–0.33]	0.14

Data are expressed as percentages, along with their 95% confidence interval (CI). None of the evaluated parameters showed a significant difference between the groups with and without follicular flushing (*p* > 0.05). Dysmorphisms include abnormalities of the zona pellucida, cytoplasm, polar bodies, endoplasmic reticulum and plasma membrane. This analysis suggests that follicular flushing does not compromise the morphological quality of oocytes.

**Table 4:** Comparative analysis of early embryonic development abnormalities between embryos derived from oocytes obtained without or after follicular flushing.

Abnormalities	Embryos from Unrinsed Oocytes (%) [95% CI]	Embryos from Oocytes After Rinsing (%) [95% CI]	*p* Value
Low Scott score 18 h post insemination by ICSI (Z3-Z4)	5.6 [4.1–7.1]	5.9 [4.4–7.4]	0.68
Embryo with BMN (multi-nuclei)	2.5 [1.3–3.7]	2.8 [1.5–4.1]	0.57
Cleavage kinetics (slow) < 4 cells at day 2 and/or <6 cells at day 3	23 [20.1–25.9]	27 [24.0–29.9]	0.04 *
Debris in embryos (fragments) >20% with unequal blastomeres	3.8 [2.5–5.1]	3.6 [2.3–4.9]	0.75
Other anomalies	1.2 [0.4–2.0]	0.9 [0.2–1.6]	0.43
Blastocysts of optimal quality (≥grade 4AA)	22.1 [24.5–31.7]	25.4 [23.9–30.9]	0.72

* Values are expressed as mean ± SD. *p* < 0.05 was considered statistically significant. Data are expressed as percentages with 95% confidence intervals. A significant difference (*p* = 0.04) was observed for slow cleavage kinetics (<4 cells at D2 and/or <6 cells at D3). No other embryonic abnormalities showed statistical variation according to oocyte retrieval mode.

## Data Availability

The data used to support the findings of this study are available from the corresponding author upon request.

## Abbreviations

The following abbreviations are used in this manuscript:

ICSI	Intracytoplasmic sperm micro-injection
COC	Cumulo-oocyte complexes
PCOS	Polycystic ovarian syndrome (PCOS).
